# Female Moth Calling and Flight Behavior Are Altered Hours Following Pheromone Autodetection: Possible Implications for Practical Management with Mating Disruption

**DOI:** 10.3390/insects5020459

**Published:** 2014-06-19

**Authors:** Lukasz Stelinski, Robert Holdcraft, Cesar Rodriguez-Saona

**Affiliations:** 1 Citrus Research and Education Center, Department of Entomology and Nematology, University of Florida, 700 Experiment Station Rd., Lake Alfred, FL 33850, USA; 2 P.E. Marucci Center, Department of Entomology, Rutgers University, 125A Lake Oswego Rd., Chatsworth, NJ 08019, USA; E-Mails: rholdcra@rci.rutgers.edu (R.H.); crodriguez@aesop.rutgers.edu (C.R.-S.)

**Keywords:** autodetection, anosmia, olfaction, pheromone communication, sex pheromone, mating disruption

## Abstract

Female moths are known to detect their own sex pheromone—a phenomenon called “autodetection”. Autodetection has various effects on female moth behavior, including altering natural circadian rhythm of calling behavior, inducing flight, and in some cases causing aggregations of conspecifics. A proposed hypothesis for the possible evolutionary benefits of autodetection is its possible role as a spacing mechanism to reduce female-female competition. Here, we explore autodetection in two species of tortricids (*Grapholita molesta* (Busck) and *Choristoneura rosaceana* (Harris)). We find that females of both species not only “autodetect,” but that learning (change in behavior following experience) occurs, which affects behavior for at least 24 hours after pheromone pre-exposure. Specifically, female calling in both species is advanced at least 24 hours, but not 5 days, following pheromone pre-exposure. Also, the propensity of female moths to initiate flight and the duration of flights, as quantified by a laboratory flight mill, were advanced in pre-exposed females as compared with controls. Pheromone pre-exposure did not affect the proportion of mated moths when they were confined with males in small enclosures over 24 hours in laboratory assays. We discuss the possible implications of these results with respect to management of these known pest species with the use of pheromone-based mating disruption.

## 1. Introduction

Insect sex pheromones are commonly used as a method of pest control with a technique termed “mating disruption” [1]. The technique takes advantage of female produced and released pheromone signals and resultant male-specific attraction to these signals by the broadcast of large quantities of synthetic pheromones into a crop or forest atmosphere from controlled release devices [1]. The technique can be highly successful; however, it is expensive and often requires application to large areas of crop for efficacy [2]. Also, some growers are unwilling to invest into a technology for which it may take several years to observe a financial return in terms of crop yield when less expensive, albeit toxic, pesticides are available as alternatives [3]. Commonly cited benefits of mating disruption include a reduction in need for pesticide input with possible benefits for the environment and less impact on non-target organisms [2,3].

Mating disruption has been investigated and improved upon over several decades with long known examples of success, but also with case studies in which the technique’s effectiveness has been highly variable [4]. Mating disruption is known to be density dependent with regard to efficacy and therefore much less effective for certain species that occur under high population densities when pesticides are not co-applied [4,5,6]. However, in certain cases, mating disruption can be highly effective in a density-independent manner [6,7]. 

Broadly, mating disruption mechanisms have been categorized as competition between synthetic point sources and authentic females [8], interference with the female’s signal that compromises male response [5,9,10], or combinations of the above [11,12]. Additional mechanisms have been suggested based on empirical research, such as advancement of the diel responsiveness of male moths to female sex pheromone following pheromone exposure [11]. This effect could potentially desynchronize male and female diel sexual responsiveness. 

Furthermore, the negative effects of delayed mating caused by mating disruption have also been recognized as significant possible contributors to population control over time following pheromone application [13,14,15,16]. Specifically, fecundity and fertility of female moths is reduced when mating is delayed by the application of synthetic pheromones. However, investigations of mating disruption have traditionally focused on the effects of pheromone on behavior and physiology of males of the target species, despite evidence that effects on female behavior may also contribute to mating disruption [17].

The capability of female insects to detect their own pheromone has been termed “autodetection”, and it is considered to occur less frequently among moth species than female anosmia (inability to detect) to their sex pheromone [18]. When autodetection is present, females are physiologically less sensitive to their pheromone than males, despite their ability to detect it [19]. In species in which females are anosmic to their pheromone, their glomeruli receiving input from pheromone-actuated sensilli are less complex than those that autodetect [19,20], and their antennae are less complex morphologically in comparison to males [18,21]. For example, autodetection is known in noctuid [22,23,24,25], yponomeutid [26], and arctiid [18] female moths, among other species discussed below.

Although it is clear that research on male moth behavior is important for practical application of sex pheromones for pest control, effects on female behavior have also proven important in several investigations. For example, pheromone exposure advances the onset of calling and proportion of calling females in tortricid moths (*Choristoneura fumiferana* (Clemens)) [27]. In some cases, pheromone exposure can increase the incidence of calling, without advancing its onset (*Cydia pomonella* (L.)) [28]. However, the effects of pheromone exposure on female moth behavior can be unpredictable and differ widely among species. For example, detection of pheromone delays the onset of calling in some species (*Adoxophyes orana* (Fischer von Röslerstamm) and *Homona magnanima* Diakonoff) [29]. However, there are also examples where autodetection does not play a role in the time of calling onset, frequency, and duration of calling in tortricids, e.g., *Pandemis limitata* (Robinson) [30], *Eupoecilia ambiguella* (Hübner), *Lobesia botrana* (Denis & Schiffermüller), and the noctuiid *Spodoptera littoralis* (Boisduval) [31].

The significance of autodetection with respect to pest management has been debated. Under high population densities, detecting pheromone may induce egg laying and subsequent dispersal as a mechanism to avoid competition [27]. Similarly, autodetection may be important for decreasing competition on limited host-plant resources under population outbreaks given that female awareness of their pheromone may contribute to dispersal flights, as a spacing mechanism [32]. In direct contrast, autodetection may also cause female aggregations as a mechanism to increase mating success [23] or result in the formation of mating leks [18]. 

As mating disruption has been developed and implemented for pest management over many decades, the effect of the sex pheromones on behavior of male insects has been studied extensively [33,34,35,36,37,38,39], but the effect on conspecific female response continues to receive less attention. With respect to pheromone exposure and subsequent response of male insects, evidence of learning, *i.e*., change in behavior as a result of experience, has been described following pheromone pre-exposure [40,41]. For example, brief pheromone exposure can counter intuitively “prime” males so that they become more responsive to pheromone several hours following exposure [40,41]. However, long-term exposure to pheromone or exposure to unnaturally high doses of pre-exposure can habituate and therefore decrease male moth response [9]. These long-term effects are also a form of olfactory learning. Although autodection by female moths is well known, as well as the broad array of possible resultant effects on female moth behavior, the possible long-term (hours) effects of pheromone pre-exposure on female moth behavior have been under-investigated. Examples of olfactory learning are not only interesting fundamentally with respect to evolutionary questions regarding population growth, but may also be relevant with respect to application of mating disruption as a pest management technique. Given that both male and female insect behavior can vary widely following pheromone pre-exposure, as described above, it is important to carry out comparative investigations with more than one species. 

In the currently described investigation, two tortricid moth species that have been historically investigated as “white rats” in mating disruption investigations (*Grapholita molesta* (Busck) and *Choristoneura rosaceana* (Harris)) were chosen to determine the long-term effects of pheromone pre-exposure on subsequent female response. Females of both moth species are known to autodetect their sex pheromone, and previous research has indicated that this may alter their diel calling cycle, total number of calling females, and egg-laying behavior [42,43]. Our specific objectives were to determine how autodetection affects female (1) calling behavior; (2) flight propensity; and (3) mating success hours following pheromone pre-exposure in order to learn more about these two species.

## 2. Experimental Section

### 2.1. Insects

*Grapholita molesta* females used in pheromone exposure experiments were from a six-year-old laboratory colony originally collected as larvae from apple orchards in southwest Michigan (U.S.). Female *C. rosaceana* moths were from an eight-year-old laboratory colony originally collected from unsprayed apple orchards in southwest Michigan. The culture was established by collecting moths as 1st and 2nd generation pupae. Both cultures were reared at 24 °C (during photophase) and 10 °C (during scotophase) and at 60% RH on pinto bean-based diet [44] under a 16:8 (L:D) photoperiod. Onset of scotophase was 18:00 hour during both moth rearing and experiments. Pupae were sorted by sex and emerged into 1-liter plastic cages containing 5% sucrose in plastic cups with cotton dental wick protruding from their lids.

### 2.2. Female Calling Behavior

This experiment tested the hypothesis that exposure to pheromone-permeated air alters the calling periodicity of female *G. molesta* and/or *C. rosaceana* moths. Virgin females of each species (2–4 days old) were placed into 1-liter plastic assay chambers (140 mm in height and 110 mm in diameter) equipped with two 0.64 cm openings in their lids as described in [42]. Glass inlets and outlets were affixed to the lids, which allowed for constant flow of carbon-filtered air (50 mL/min) through the chambers. Carbon-filtered (Model 100 Safe Glass Hydrocarbon Trap, Chromatography Research Supplies, Louisville, KY, USA) air entering chambers was passed through 1-liter flasks containing rubber septa loaded with the pheromone of each respective species. For *G. molesta*, septa were loaded with 0.01 mg of a three-component blend of: (*Z*)-8-dodecenyl-acetate:(*E*)-8-dodecenyl-acetate:(*Z*)-8-dodecenol in a 93:6:1 ratio (Shin-Etsu Chemical Co., Ltd., Tokyo, Japan, confirmed by Gas Chromatography). This blend and pheromone dosage is attractive to *G. molesta* males [41], and is known to affect calling behavior of females [42]. 

For experiments with *C. rosaceana*, carbon-filtered air entering chambers was passed through 1-liter flasks containing rubber septa loaded with 0.05 mg of (*Z*)- and 0.002 mg of (*E*)-11-tetradecenyl acetates and 0.003 mg of (*Z*)-11-tetradecenol. This blend and dosage were used because of known attractiveness to males [45]. The specific dosage was the lowest tested to consistently alter female calling behavior in preliminary experiments. It is also a dosage that may likely influence mating disruption of this species [43].

Assay chambers were housed in a Plexiglas flight tunnel similar to that detailed in [42]. Temperature and photoperiod were controlled (16:8 L:D) and maintained at 23 °C and 50%–70% RH. Light intensity inside the tunnel during photophase was 1000–1500 lux, and was generated by two fluorescent bulbs mounted 10 cm above the flight tunnel. During scotophase, light intensity was *ca*. 3–10 lux likely due to lack of complete darkness in the assay room and with no intended light source present. Air was pushed through the tunnel by an upwind fan at 0.3 m/s, and pheromone emerging from assay chambers was expelled from the tunnel and building through a wall-mounted pipe for evacuation of chemicals from the experimental chamber. 

**Figure 1:**
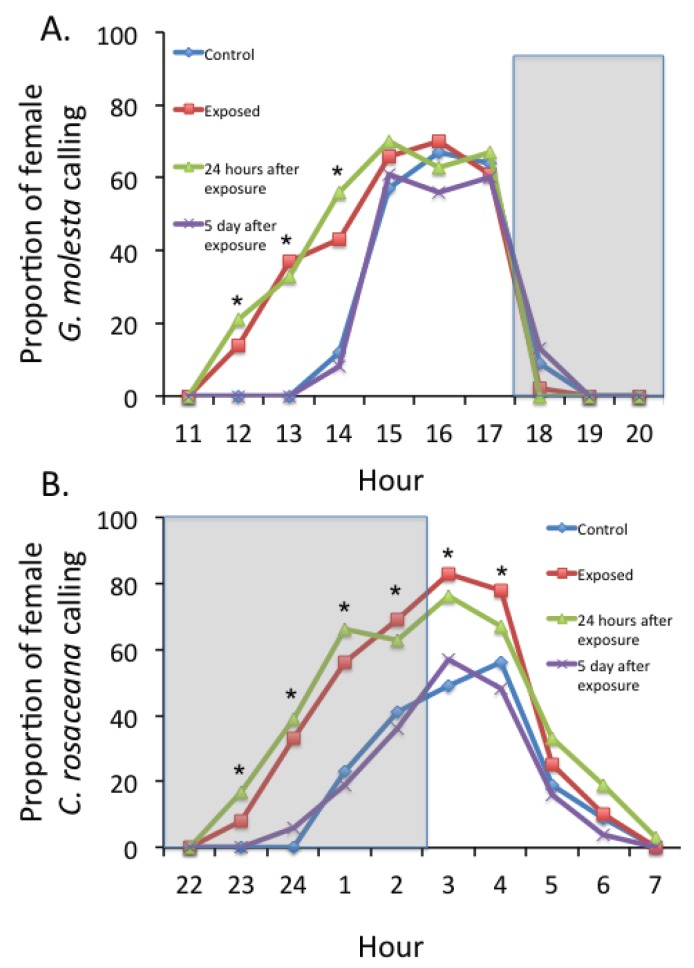
Proportion of female *Grapholita molesta* (**A**) or *Choristoneura rosaceana* (**B**) calling throughout the diel cycle under no pheromone exposure, during pheromone exposure, and 24 hours or 5 days following pheromone exposure. Significant (*p* ≤ 0.05) differences between the proportions of female moths calling in the pheromone treatments are depicted by “*”. Shaded gray areas depict scotophase. *n* = 32.

The experimental setup is depicted in Figure 1 of [42]. The experiment was repeated on eight different days for each species and pheromone exposure treatment, and replicated with four assay chambers per treatment on each day. The four assay chambers were observed simultaneously on each day. The treatments were: (1) control chambers that received air input from flasks containing septa loaded with hexane solvent only; (2) chambers that received constant air from flasks that contained pheromone-treated septa; (3) exposure to clean air 24 hours after 10 hours of pre-exposure to pheromone contaminated air as in treatment 2; (4) exposure to clean air for 5 days after 10 hours of pre-exposure to pheromone-contaminated air as in treatment 2. Ten hours of pre-exposure was chosen because that interval approximately bracketed one day of possible female calling behavior for each species (see Figure 1). During the interval between pre-exposure and testing, females were housed in pheromone-free rearing chambers under the conditions described above. It is possible that female moths received some level of pheromone auto-exposure during culture maintenance and prior to experiments, as they were held in close proximity within rearing cages. However, this occurred in all females including those in the control treatment and thus did not likely affect our ability to compare female behaviors among treatments.

For *G. molesta*, five virgin females were placed into each treatment chamber per replicate. Females were acclimated in chambers for 30 min prior to initiating observations. The experiment commenced at 10:30 hour and was terminated at 20:30 hour. The number of female *G. molesta* observed calling was recorded hourly according to the criteria for female calling behavior described by [46]. During scotophase, observations were conducted with the aid of night-vision goggles described by [47]. Experiments with *C. rosaceana* were conducted identically, except that the experiment commenced at 20:00 hour and was terminated at 8:00 hour; onset of scotophase was 18:00 hour. The number of females observed calling was recorded hourly. Female calling was quantified by counting the number of females assuming a posture characterized by raised wings and a protruding abdomen as has been observed with other tortricids [30,46]. Experiments were conducted separately for each species.

### 2.3. Flight Mill Testing Following Pheromone Pre-Exposure

This experiment tested the hypothesis that exposure to pheromone-permeated air alters the flight behavior of female *G. molesta* and/or *C. rosaceana* moths. Mated, 2–4 days old female *G. molesta* or *C. rosaceana* were pre-exposed to pheromone in 1-liter plastic assay chambers housed in a wind tunnel as described above. To establish treatments, carbon-filtered air was pushed (50 mL/min) through the assay chambers via 1-liter flasks as described above. Female exposure treatments lasted 10 hours, *i.e*., the period equivalent to maximal possible calling behavior per diem. Female moth behavior for each species was assayed after the following treatments: (1) no exposure (control); (2) 24 hours after 10 hours of pre-exposure to pheromone-contaminated air as described above; (3) 5 days after 10 hours of pre-exposure to pheromone-contaminated air as described above (Table 1).

**Table 1:** Effect of sex pheromone exposure on female flight of *Grapholita molesta* and *Choristoneura rosaceana* on a laboratory flight mill 24 hours or 5 days following prolonged auto-exposure.

Species	Treatment	Number of moths tested	Proportion of moths responding ^1^	Mean Flight duration ± SE (min) ^1^
*G. molesta*	Control	50	0.66 b	315.9 ± 45.3 b
	24 hours after exposure	50	0.90 a	544.3 ± 82.0 a
	5 days after exposure	50	0.74 ab	246.5 ± 32.2 b
*C. rosaceana*	Control	50	0.68 b	166.0 ± 26.1 b
	24 hours after exposure	50	0.86 a	537.0 ± 140.6 a
	5 days after exposure	50	0.74 ab	140.5 ± 30.4 b

^1^ Within each moth species, different letters within a column indicate significant differences among treatments (*p* ≤ 0.05).

Female flight behavior was investigated following pheromone pre-exposure. Fifty moths were tested per species and per treatment. All females tested were unmated and 2–3 days old prior to treatment. The flight behavior of female *G. molesta* has been described in detail previously [48], and this moth age and mating status is appropriate for testing of optimal flight behavior. A similar previous investigation with *C. rosaceana* warranted use of moths of the same age and mating status for this species [49]. All flight mill assays were conducted under the rearing conditions described above. The flight mill consisted of a simple horizontal rotor that was attached to a vertical pivot and which was suspended between two small magnets as described in previous similar assays [48,49]. The distance between the axis and moth attachment point was 15.25 cm (95.8 cm revolution circumference). Flights were assayed under the moth-rearing conditions described above at approximately 24 °C.

Female moths of each species were immobilized with exposure to carbon dioxide prior to attachment to the flight mill rotor. Preliminary testing showed no adverse effects of this treatment on flight capability. Following immobilization, scales from the dorsal mesothorax were removed with a fine camel hair brush, and approximately 0.03 mg of Elmer’s craft glue (Columbus, OH, USA) was applied to the exposed cuticle. Each moth was attached to a fine silver wire harness by direct contact with glue for 30–40 s. Each harness with a connected moth could be easily attached to the flight mill by inserting it into a syringe-type opening in the rotor blade. A complete flight by a moth was considered when it lasted at least 20 s, making approximately 6–10 rotations. Following the pheromone pre-exposure treatment, the proportion of moths initiating flight and the total duration of flight per moth were quantified for each species separately. All data were collected using manual counters with direct observation.

### 2.4. Effect of Pheromone Pre-Exposure on Mating Frequency

The effects of pheromone pre-exposure on mating frequency hours or days after exposure were investigated. Unmated, 2–4 days old female *G. molesta* or *C. rosaceana* were placed in 1-liter plastic assay chambers and housed within a wind tunnel as described above. Carbon-filtered air was pushed (50 mL/min) through the assay chambers via 1-liter flasks establishing the following treatments: (1) exposure to clean air; (2) exposure to clean air 24 hours after 10 hours of pre-exposure to pheromone contaminated air as described above; (3) exposure to clean air 5 days after 10 hours of pre‑exposure to pheromone contaminated air as described above (Figure 1). The experiment was replicated on three different days for each pheromone exposure treatment tested with 5 assay chambers containing 5 virgin female *G. molesta* or *C. rosaceana* per treatment replicate. The pre-exposure treatment was randomized daily. Ten *G. molesta* or *C. rosaceana* males were placed into each replicate chamber during the experimental assay. The experiment ran for 24 hours with a 16:8 (L:D) photoperiod after which the total number of mated females was determined by dissection of the *bursa copulatrix* revealing the presence or absence of a spermatophore as described in [50].

### 2.5. Statistical Analyses

Differences between the proportions of female *G. molesta* calling for each hour of observation in pheromone-treated *versus* control chambers were analyzed using the Kruskal-Wallis test followed by Mann-Whitney U tests with a Bonferroni corrected 0.05 alpha value [51]. Differences in proportions of female moths responding between treatments in the flight mill tests following pheromone pre-exposure were tested with logistic regression and significance was determined using the log-likelihood ratio χ^2^ (The LOGISTIC Procedure, SAS 9.1) [51]. Moths that did not exhibit flight following tethering and treatment application were not considered in this analysis. A sufficient number of moths was obtained to achieve an *n* = 50 per treatment. Flight duration data were subjected to analysis of variance (ANOVA) following log transformation to homogenize variance, and differences between means were separated using Tukey’s multiple comparison tests [51]. A logistic model was used to measure the probability of mating by moths of each species with regard to pheromone pre-exposure treatments. Proportion of moth mating was analyzed using the G statistic [52] with the PROC GENMOD procedure in SAS (Statistical Analysis System) [51], and in all cases differences between means were considered significantly different at the α < 0.05 level. 

## 3. Results

### 3.1. Effect of Pheromone Pre-Exposure on Female Calling

The calling behavior of *G. molesta* in the control treatment began soon after hour 13:00 and terminated after hour 17:30, which was soon after the onset of scotophase (Figure 1A). Female *G. molesta* that were pre-exposed to their own pheromone components 24 hours prior, initiated calling approximately 2 hours earlier than control females (χ^2^ = 97.5, df = 2, *p* < 0.001) (Figure 1A). The earlier onset of calling behavior observed in females that were pre-exposed to pheromone 24 hours earlier was essentially indistinguishable from that observed with females that exhibited an accelerated onset of calling when under a direct stream of pheromone exposure (Figure 1A). The proportion of females calling at 15:00–17:00 hour was not different between pheromone pre-exposed (24 hours and 5 days prior), directly pheromone-exposed, and control treatments (*p* > 0.05); and time of calling termination did not differ between treatments (*p* > 0.05) (Figure 1A). The calling behavior of female *G. molesta* 5 days after pheromone pre-exposure was not different from that observed with non-exposed controls (*p* > 0.05).

For female *C. rosaceana*, calling commenced after hour 22:00 and terminated after hour 6:00 (Figure 1B). Both the pheromone pre-exposure treatment 24 hours prior to assays and direct pheromone exposure during assays significantly accelerated female calling by approximately one hour (χ^2^ = 97.5, df = 2, *p* < 0.001) (Figure 1B). Also, both pheromone pre-exposure and direct pheromone exposure significantly increased the total proportion of *C. rosaceana* females that exhibited calling behavior as compared with the clean air control (*p* ≤ 0.05) (Figure 1B). However, calling behavior of females 5 days after pre-exposure was not different from females observed in the no exposure control treatment (*p* > 0.05) (Figure 1B).

### 3.2. Effect of Pheromone Pre-Exposure on Female Flight

A significantly greater proportion of *G. molesta* females initiated flight 24 hours, but not 5 days, after pheromone pre-exposure as compared with non-exposed controls (Table 1). Also, 24 hours after exposure, female *G. molesta* flew, on average, for a significantly longer duration than unexposed controls and those females that were assayed 5 days following pheromone pre-exposure. There was no statistical difference in the proportion of *G. molesta* females initiating flight and total duration of flight between the unexposed control treatment and females that were tested 5 days following pheromone pre-exposure (Table 1).

The flight mill results with female *C. rosaceana* were similar to those observed with *G. molesta* (Table 1). More female *C. rosaceana* initiated flight 24 hours after pheromone pre-exposure and flew longer than the unexposed control treatment; however, there was no statistical difference in behavior between control females and those that were 5 days following pheromone pre-exposure (Table 1).

### 3.3. Effect of Pheromone Pre-Exposure on Mating Success

**Figure 2:**
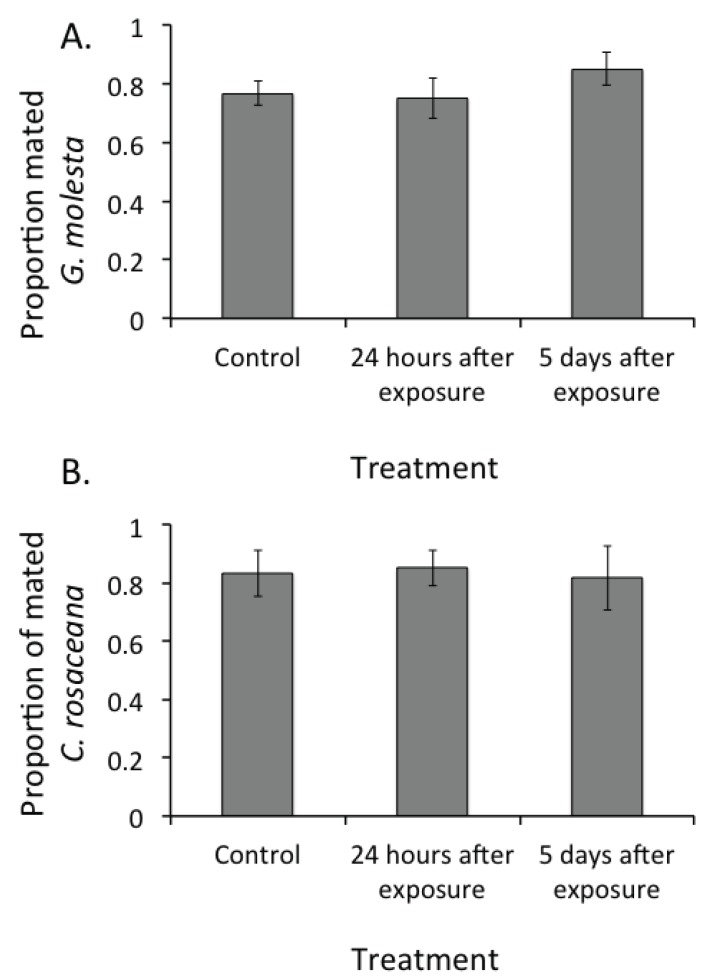
Mating status of female *Grapholita molesta* (**A**) or *Choristoneura rosaceana* (**B**) following a 24 hours interval in confined 1-L cages with a conspecific male under no pheromone exposure, and 24 hours or 5 days following pheromone exposure.

At pheromone dosages that affected both calling and flight behavior of *G. molesta* and *C. rosaceana*, pheromone pre-exposure did not affect the proportion of mated *G. molesta* (Figure 2A) or *C. rosaceana* (Figure 2B) at either 24 hours or 5 days following pre-exposure as compared with the unexposed control treatment when pre-exposed or control females were confined with males (*p* > 0.05).

## 4. Discussion

Our results indicate that autodetection in two species of tortricids can result in learning for a significant duration after exposure to pheromone. Learning is often defined as a change in behavior following previous experience with a stimulus [40,41]. This occurred in two species of tortricid moths, which are both agricultural pests of tree fruit crops. Both of these species are known for autodetection as measured electrophysiologically, and in both species, direct exposure to pheromone is known to alter female calling behavior [42,43]. Specifically, calling behavior is advanced during pheromone exposure in *G. molesta* [42], and both advanced and overall increased in *C. rosaceana* [43]; both effects were confirmed in the current study. Autodetection is also known to advance onset of calling and increase the total proportion of calling in the tortricid, *C. fumiferana* [27]. Similarly, for the tortricid, *C. pomonella*, autodetection increases the incidence of calling behavior; however, in this case, it does not advance its onset [28]. In the current investigation, we show that this effect persisted for 24 hours, but not for 5 days, after 10 hours of pre-exposure. However, our experiment did not rule out the possibility that moth aging may have contributed to this reduced responsiveness at 5 days following pre-exposure. Although the precise duration of exposure required for this learning to occur, as well as, the total duration of behavioral change following learning was not quantified, we document that learning is maintained for at least 1 day. However, by 5 days after exposure, the previously learned response was mostly abolished. The duration of pre-exposure in this case was chosen based on the approximate time that a female tortricid moth may be exposed to synthetic pheromone applied for mating disruption during her possible daily window of calling behavior in the field. However, it cannot be ruled out that long durations of pre-exposure habituate female response as does occur with males [9]. Under mating disruption, where pheromone is continuously applied to the crop, in some cases for 100 or more days [2], we presume that the propensity for pheromone exposure and this type of learned response may be significant, depending on the average airborne pheromone dosage of pre-exposure. 

The possible practical significance of autodetection and its long-term effects on female calling behavior is de-synchronization of female calling and associated male response. Such a mechanism has been postulated previously for male moth response following pheromone exposure [11]. Specifically, if autodetection and possible long-term advancement of female calling following pheromone exposure occurs in the field, it could possibly de-synchronize the window of female calling behavior with the overlapping window of male moth responsiveness to their conspecific sex pheromone. Biologically, these windows of female calling and male moth response are modulated by endogenous circadian rhythms that are affected by exogenous factors such as temperature [46]. There is a likely evolutionary advantage to such environmentally mediated circadian cycles given that these moth species occur over multiple (2–3) generations in temperate climates where weather is colder in the spring and warmer in the summer [46]. However, the input of synthetic pheromone for mating disruption may affect these rhythms that are naturally calibrated by abiotic climate factors. Determining the effects of autodetection on practical management of tortricid moths and other species will benefit from further field-oriented investigations.

Pheromone pre-exposure for both moth species tested here also affected subsequent moth flight behavior, as measured in the laboratory, for a significant duration following exposure. The time intervals for behavioral assays following exposure were chosen based on the typical span of optimal moth oviposition behavior for these species [15]. For both species, the propensity for initiating flight and the total duration of flight increased 24 hours after autodetection-based pre-exposure to their own sex pheromone. Although there was some evidence that the proportion of moths initiating flight was higher for up to 5 days after pheromone exposure, these responses were no longer statistically different from the unexposed controls (Table 1). It is possible that moth age may have been a factor in this experimental design and that older moths were poorer fliers than the younger ones given the four-day difference in age between treatments. This should be addressed in subsequent investigations. 

It has been hypothesized that autodetection may be a mechanism that reduces competition between conspecific females, under high population densities, given that females may detect pheromone-based competition for males [27]. A complementary hypothesis is that autodetection may be a spacing mechanism among females that are competing for limited host-plant resources [32]. The current results suggest that, depending on the level of pheromone auto-exposure, females may maintain this behavior for several hours as a learned response, which would be advantageous to minimize competition among limited resources, whether these resources are conspecific males or oviposition sites. From a practical perspective, this may be a mechanism that causes female moths of certain species to disperse under deployment of synthetic pheromones for mating disruption. This may cause infestation of neighboring unprotected areas and may also increase the likelihood for subsequent re-infestation of the pheromone-treated points of origin if moths disperse between treated and untreated orchards [47]. This may be one contributing reason for the necessity of large-scale, area-wide pheromone application for effective management of tortricid moth pests with mating disruption [2,3]. 

In the laboratory experiments conducted here, autodetection did not affect female mating, when they were paired with males in confined (1-L volume) laboratory enclosures. This assay prevented female dispersal and allowed male access to apparently receptive females in a confined space. These results indicate that pheromone pre-exposed females are equally capable of mating as compared with non-exposed controls, despite that such exposures advance female calling time and increase their flight propensity and duration. More sophisticated assays or field tests are suggested to determine how the effects of auto-exposure and associated learning in females may affect female mating in nature or under synthetic pheromone treatments to protect crops by mating disruption.

## 5. Conclusions

Mating disruption is perhaps the most successfully used semiochemical-based technique worldwide for management of pests [1]. The technique has been investigated for decades and is in some cases implemented in commercial agriculture on very large scales that rival traditional insecticide use [2,3]. The mechanisms of mating disruption were a subject of great interest at the infancy of the technique’s development, but focused on male moths almost exclusively [9]. Fundamental investigations of moth chemoreception have revealed female autodetection [18,21]. Several hypotheses (described above) have been proposed to explain the possible evolutionary benefits of autodetection to reduce female-female competition among limited resources. As mating disruption has gained ground as a useful pest management tool worldwide [1], it is possible that autodetection, which may have evolved to reduce female–female competition and potentially increase individual female fecundity and overall population growth, may now be a mechanism that increases females’ capability of evading mating disruption. Significantly more laboratory and field testing is necessary to test this hypothesis. Also, it is possible that area-wide use of mating disruption already mitigates the possible behavioral effects of autodetection on pest management with sex pheromones.
